# Induction of Gametogenesis in the Cnidarian Endosymbiosis Model *Aiptasia* sp.

**DOI:** 10.1038/srep15677

**Published:** 2015-10-26

**Authors:** Désirée Grawunder, Elizabeth A. Hambleton, Madeline Bucher, Iliona Wolfowicz, Natascha Bechtoldt, Annika Guse

**Affiliations:** 1 Centre for Organismal Studies (COS), Heidelberg University, Heidelberg 69120, Germany; 2 University of Porto, Porto 4200-465, Portugal

## Abstract

Endosymbiosis is widespread among cnidarians and is of high ecological relevance. The tropical sea anemone *Aiptasia* sp. is a laboratory model system for endosymbiosis between reef-building corals and photosynthetic dinoflagellate algae of the genus *Symbiodinium*. Here we identify the key environmental cues to induce reproducible spawning in *Aiptasia* under controlled laboratory conditions. We find that simulating a lunar cycle with blue-wavelength light is necessary to promote abundant gamete production and synchronous release in well-fed animals. Sexual reproduction rates are genetically determined and differ among clonal lines under similar conditions. We also find the inverse difference in rates of asexual reproduction. This study provides the requisite basis for further development of the *Aiptasia* model system, allowing analysis of basic cellular and molecular mechanisms in the laboratory as well as investigations of broad questions of ecological and evolutionary relevance.

Cnidarians exhibit enormous plasticity in their morphologies and life cycles, and have emerged as key models in a broad range of research fields from evolution to ecology. As the sister group to the Bilateria (Fig. 1a), cnidarians are important model systems for studying development^1^,^2^, pattern formation^3^, regeneration^4^ and stem cell biology^5^,^6^ (Fig. 1a). The cnidarians’ evolutionary success over 500 million years has been proposed to be due in part to their ability to form symbioses with prokaryotic and/or eukaryotic microorganisms^7^. Major symbiotic partners include complex communities of viruses, archaea, bacteria (including cyanobacteria), and eukaryotic algae^8^,^9^. More broadly, such symbioses with photosynthetic microbes are also found in mollusks, sponges, acoel flatworms, and vertebrates (salamander) and in all, the translocation of photosynthetically fixed carbon from the symbiont to the host represents a significant energy source^10^,^11^.

The most common eukaryotic endosymbiont among the cnidarians is the dinoflagellate *Symbiodinium* spp.; associations with *Symbiodinium* are found in many species including the majority of hexacorallia (e.g. reef-building corals, sea anemones), octocorallia (e.g. gorgonians, soft corals, sea pens), hydrozoa (e.g. fire corals) and scyphozoa (e.g. jellyfish)^12^ (Fig. 1a). Genus *Symbiodinium* is diverse, containing hundreds of strains worldwide that have been categorized into clades based on ribosomal DNA markers^13^; species assignments and functional characterizations of strains are an active area of research^14^. The unicellular *Symbiodinium* reside intracellularly in cnidarian endodermal tissues and transfer photosynthates to the host. Thus, this widespread phenomenon is likely a key factor in the extensive adaptive radiation of cnidarians in diverse aquatic niches.

The most economically and ecologically critical cnidarian-*Symbiodinium* symbiosis is that of reef-building corals, which rely so heavily on symbiont-produced nutrition that the relationship is obligatory for the hosts to persist^15^,^16^. As such, the breakdown of this symbiosis, termed “coral bleaching”, has become a major threat to coral reefs worldwide^17^. Despite this importance, much remains unknown about the cellular and molecular basis of the coral-*Symbiodinium* symbiosis, including its establishment, maintenance, and breakdown in response to stress^18^. The majority of corals produce symbiont-free planula larvae that must take up symbionts from the environment each generation^19^. However, most reef-building corals spawn only once per year^20^, greatly restricting the experimental availability of larvae and thereby severely limiting the systematic study of endosymbiosis establishment.

To address this limitation, a practicable symbiotic laboratory model has been developed with the small sea anemone *Aiptasia* sp. Although *Aiptasia* is not suited to every application relevant to coral biology (e.g. calcification), it holds many advantages including its symbiotic relationship with the same types of *Symbiodinium* strains as corals^21^,^22^,^23^. Most importantly, the process of endosymbiosis establishment is similar to that of many reef-building corals: *Aiptasia* planula larvae are initially non-symbiotic and establish endosymbiosis anew each generation^23^ (Fig. 1b). Both reef-building corals and *Aiptasia* live primarily as a sessile polyp stage that switches between asexual reproduction and sexual production of motile planula larvae (Fig. 1b). Based on molecular analysis, *Aiptasia* sp. is regarded as a single panglobal species with two distinct genetic networks: one on the United States South Atlantic coast and the other consisting of all other *Aiptasia* sp. sampled worldwide^24^. Clonal anemone lines can be generated through asexual reproduction via pedal laceration^25^ (Fig. 1b), and the majority of *Aiptasia* resources have been developed from clonal line CC7, including transcriptomes^26^,^27^ and the genome^28^. Transcriptomic and genomic resources for many *Symbiodinium* strains are likewise available^29^,^30^,^31^.

Despite these advantages, a critical aspect remains underdeveloped in *Aiptasia*: consistent induction of sexual reproduction in the laboratory, a prerequisite to investigations of symbiosis establishment and development of state-of-the-art functional tools such as morpholinos and transgenesis^32^,^33^,^34^. *Aiptasia* is demonstrably capable of year-round gametogenesis^35^ and spawning in laboratory conditions^23^,^36^, but laboratory-induced spawning has been inefficient and unpredictable and currently no publically available protocols exist to overcome this limitation. Laboratory-induced spawning in the cnidarian *Nematostella* has been achieved through manipulation of food, temperature, and light alone or in combination^37^,^38^. In the wild, coral spawning is correlated with the lunar cycle and blue light appears to play a major role in synchronized spawning^39^,^40^,^41^,^42^. Similarly, oogenesis in *Aiptasia* is increased by feeding^43^ and peaks in relation to the lunar cycle^35^. Thus, we hypothesized that ample feeding and blue-light cues simulating moonlight may efficiently stimulate laboratory spawning in *Aiptasia*.

To test this hypothesis, we used three *Aiptasia* clonal lines used in laboratories worldwide. We first further characterized these clonal lines—CC7 (male) and F003 and H2 (both female)—and used molecular phylogeny to place them into a biogeographical context. Next, we systematically tested different conditions to determine major cues for coordinated and copious gametogenesis and spawning. We found that blue-light cues (presumably mimicking a lunar cycle) together with slight temperature increases produce synchronous and efficient spawning in well-fed CC7 and F003 couples and, to a lesser extent, in CC7 and H2 couples. Quantitative differences of spawning efficiencies between female lines F003 and H2 were inversely correlated to their asexual reproduction rates.

## Results

### Characterization of *Aiptasia* clonal lines

The three clonal lines of *Aiptasia* CC7, F003, and H2 we routinely maintain in the laboratory (see *Aiptasia* culture conditions in Methods) appear morphologically similar (Fig. 2a). However, their genetic relationship to each other and to other *Aiptasia* populations was unclear. We therefore determined the relationship of our lines to wild *Aiptasia* populations worldwide by sequencing the four independent “*SCAR”* (“sequence characterized amplified region”) genotyping markers^24^ of two individuals from each of the three clonal *Aiptasia* lines. Within each of the lines, both individuals sampled yielded identical sequences for each *SCAR* marker, demonstrating the continued clonality of the lines. Each clonal line produced a haplotype distinct from the others. In CC7 and F003, two alleles were found in the following three loci and both animals carried the alleles: *SCAR3*, *SCAR4*, and *SCAR5* (CC7 only). In those cases, the alleles were collapsed together to produce a consensus sequence containing the appropriate base ambiguity. The Maximum Likelihood tree in Fig. 2b shows the relationship of these three clonal lines to twelve globally field-sampled *Aiptasia* representing eight distinct haplotypes^24^. As expected, lines CC7 and F003 (founders collected from US South Atlantic coast) and line H2 (founder collected from Hawaii) all cluster closest with individuals collected from the same respective geographic region by Thornhill and colleagues^24^ (Fig. 2b). Thus, these laboratory clonal lines represent members of both identified *Aiptasia* genetic networks found worldwide: US South Atlantic network (CC7, F003) and global network (H2).

### Endogenous *Symbiodinium* hosted by *Aiptasia* clonal lines

Thornhill and colleagues^24^ found that the two worldwide *Aiptasia* genetic networks differ in their symbiont composition: the global *Aiptasia* network harbors only the clade B species *Symbiodinium minutum*, while members of the US South Atlantic network harbor clade A *Symbiodinium* strains either solely or, to a lesser extent, in combination with either variants of clade B *S. minutum* or (rarely) clade C strains (Fig. 2b). Accordingly, clonal line H2 (global network) harbors only the *S. minutum* strain SSB01^22^, while clonal line CC7 (US South Atlantic network) hosts a single clade A strain^26^ designated SSA01 (Fig. 2b). To identify the as-yet-unknown endogenous symbiont(s) of *Aiptasia* clonal line F003 (US South Atlantic network), we sequenced the chloroplast ribosomal 23S subunit (*cp23S*) of three independent F003 animals as previously described^22^. Within each individual, we consistently found three distinct *cp23S* sequences in relatively constant proportions: ~57% of sequences were a single variant of *S. minutum* (i.e. identical to strain SSB01 [GenBank #JX221048.1] except for a SNP at SSB01 nt250 and a deletion in SSB01 nt363–nt498); ~35% were identical to SSA01 (GenBank #KT186239); ~8% were identical to *S. minutum* strain SSB01. The first two sequences are consistent with the typical patterns of the US South Atlantic *Aiptasia* network^24^. The third sequence, *S. minutum* strain SSB01, was not detected during a similar but cursory analysis >1.5 years ago; its current presence may be due to either its previously undetected natural occurrence or to an accidental introduction during extended culture in close proximity to SSB01. Taken together, F003 and CC7 are therefore each representatives of different symbiont patterns typically observed in the US South Atlantic *Aiptasia* network.

### Spawning induction for *Aiptasia* clonal lines CC7, F003, and H2 under laboratory conditions

To test whether the simulation of a lunar cycle can trigger spawning in *Aiptasia*, we subjected spawning couples to a blue-intensive moon cue for five nights per 29-day cycle (Fig. 3a–c and Supplementary Fig. S1 online). Beforehand, animals were fed with *Artemia* nauplii five times per week for over two months. To avoid competition for food and other resources, animals were cultured in low densities and pedal lacerates were removed on a regular basis (we observed that such treatment markedly increases animal size [Supplementary Fig. S2 online]). We monitored in total 13 couples of [F003xCC7] and 37 couples of [H2xCC7] for two subsequent cycles (Fig. 3a–c). For crosses with F003, spawning was tightly clustered between Day 13 and Day 20 of the 29-day artificial lunar cycle, with peak larvae production on Days 13–17 (Fig. 3b). Spawning in crosses with H2 was likewise synchronous, with a peak of larvae production also around Day 13–18 (Fig. 3c). Overall, couples with F003 were more synchronous and spawned more often than those with H2 (Fig. 3b,c) (see below for more on observed line-specific differences). In both lines, however, the variability of larvae occurrences between couples was relatively high, which may be due to differences in culture conditions prior to spawning induction (Supplementary Fig. S1 online). We monitored couples for only two spawning cycles because spawning frequencies decreased afterwards, somewhat depending on the duration in the pre-induction high-feeding conditions (data not shown).

Because we sought to modify our spawning protocol to maximize spawning efficiency and predictability, during these experiments we intermittently tested other conditions previously known to stimulate spawning in other cnidarians. We compared spawning when animals were fed *Artemia* nauplii twice weekly versus five times per week; we observed that increased feeding improved spawning, especially for F003-containing couples (Supplementary Fig. S3 online). We tested whether a very low amount of blue light during the pre-spawning-induction period (see Methods) would enhance spawning efficiency during the lunar cycle inductions, yet saw no discernable effect of these treatments (Supplementary Fig. S1 online). Because warming water temperatures appear correlated with gametogenesis and spawning in anthozoans^36^,^38^,^39^,^44^, we simultaneously tested the effects of temperature shifts during spawning induction. We saw no obvious trend, but interpretation was difficult because the animals in the experiments were subjected to different conditions prior to spawning induction (Supplementary Fig. S1 online). We therefore sought to parse which cues were key to promote efficient and synchronous spawning.

### Blue light is the key cue to induce efficient and synchronous spawning to produce larvae

To determine the distinct effects of temperature and simulated moon cue to effectively induce spawning, we conducted a side-by-side comparison of different spawning cues using only couples of [F003xCC7] originating from identical pre-induction culture conditions. Couples were grown at 26 °C and fed well for seven months prior to exposure to the following conditions: (A) blue-light simulated moon at 29 °C; (B) blue-light simulated moon at 27 °C; (C) white-light simulated moon at 29 °C; (D) no simulated moon (Fig. 3d). Blue light appeared to be the most important cue, as these conditions (A and B) produced efficient and synchronized spawning of larvae (Fig. 3e) as well as copious production of tens of thousands of larvae (Fig. 3f). The temperature increase from growth conditions to 29 °C (Conditions A, C, and D) appeared to be beneficial to egg release (Fig. 3e), although this temperature shift alone or with a white-light cue results in inefficient larvae production, with either no or very low numbers of larvae generated (Fig. 3f). White light, which in this case is composed of ~20% blue wavelengths (see Methods), does stimulate some spawning, but mostly eggs (Condition C, Fig. 3e) and over a wider range of days in the cycle (Days 16–21, Supplementary Fig. S4 online). This poor synchronization and low output resulted in very little larvae, especially when compared to condition A in which the only difference is the blue spectrum of the light (Fig. 3f). All data for these experiments are found in Supplementary Fig. S4 online.

### Quantification of line-specific differences in larvae production

To confirm and quantify the differences we observed in spawning efficiencies between females of clonal lines F003 and H2 (Fig. 3b,c), we performed additional experiments under the best spawning induction parameters from above. We chose F003, H2, and CC7 animals of similar body size and cultured them in low-density cultures that were fed five times a week for five months, after which five couples of [F003xCC7] and five couples of [H2xCC7] were subjected to the blue-light lunar cycle cue (Condition A, Fig. 3d) and larval output was quantified. Consistent with our observations in the earlier experiments (Fig. 3b,c), we again found that couples with F003 spawned much more efficiently than those with H2: crosses with F003 produced larvae on average 1.8 times per couple per cycle, whereas H2 produced larvae only an average of 0.2 times per couple per cycle (Fig. 4a). Reproductive output for F003 was also far higher, typically with over 10,000 larvae per event, than for H2-containing couples that only spawned small numbers of larvae or, in most cases, eggs (Fig. 4a). Nevertheless, spawning synchrony was high in crosses with both female lines, falling between Days 13 and 18 (Fig. 4a).

### Line-specific differences in gonad development

In *Aiptasia* sp. gonad development occurs within the mesentery tissue in the interior body column and is directly dependent upon food uptake^43^. We dissected well-fed CC7, F003, and H2 animals and indeed found many gonads within mesentery tissue (Fig. 4b, upper row). CC7 male gonads were typically smaller and lighte167 r in comparison to the bigger, darker gonads from females in lines F003 and H2 (Fig. 4b, lower row). However, qualitative visual assessments consistently showed that F003 animals contained higher ratios of distinct gonads to non-gonad mesentery than H2 animals (Fig. 4b). In both F003 and H2 gonads, we observed immature oocytes with prominent germinal vesicles, in contrast to released eggs during spawning (Supplementary Fig. S5 online). Sperm isolated from CC7 gonads were morphologically similar to those naturally released during spawning (Supplementary Fig. S5 online).

### Line-specific differences in asexual reproduction

**Figure 1:**
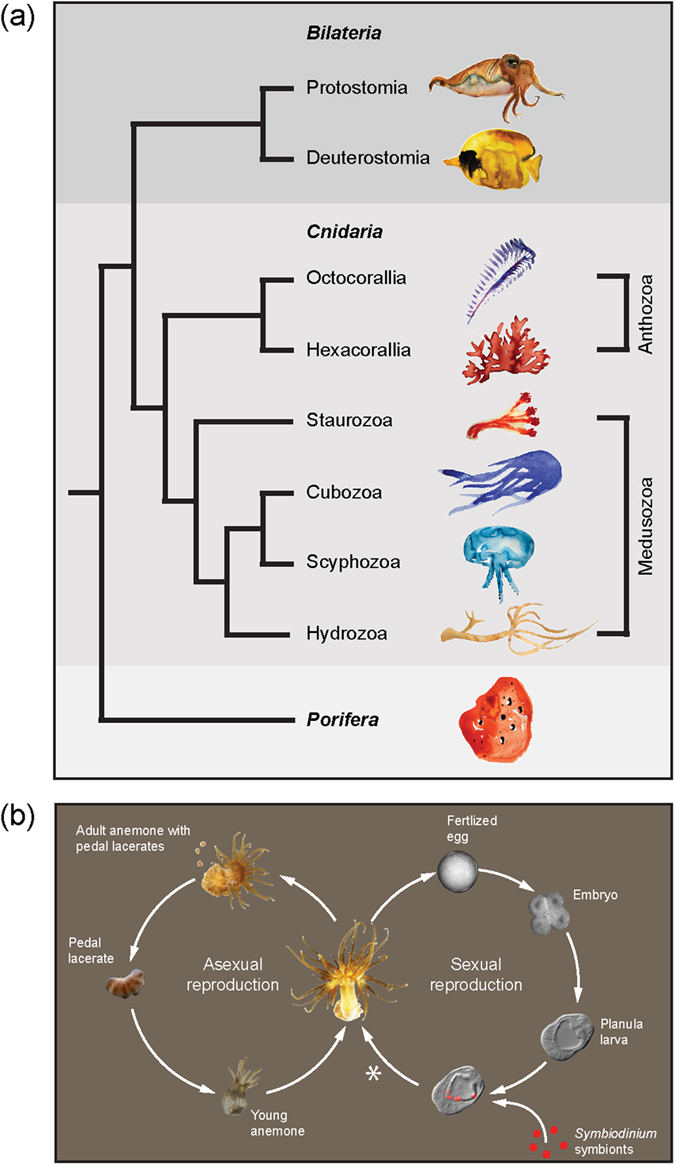
Symbiosis throughout the cnidarians and the anthozoan life cycle. (**a**) Phylogenetic tree of the major metazoan clades, with phyla shown in bold. Cnidarians represent a sister group to the bilaterians and are at the base of metazoan evolution. Symbiosis with dinoflagellates and green algae occur in species throughout the classes Medusozoa and Anthozoa, the latter of which contains *Aiptasia* sp. Illustrations were drawn by Stephanie Guse and are used with permission. (**b**) Overview of *Aiptasia* life cycle showing dual reproductive modes. *metamorphosis and settlement in the laboratory have not yet been reported, and as such remains an active experimental area.

**Figure 2:**
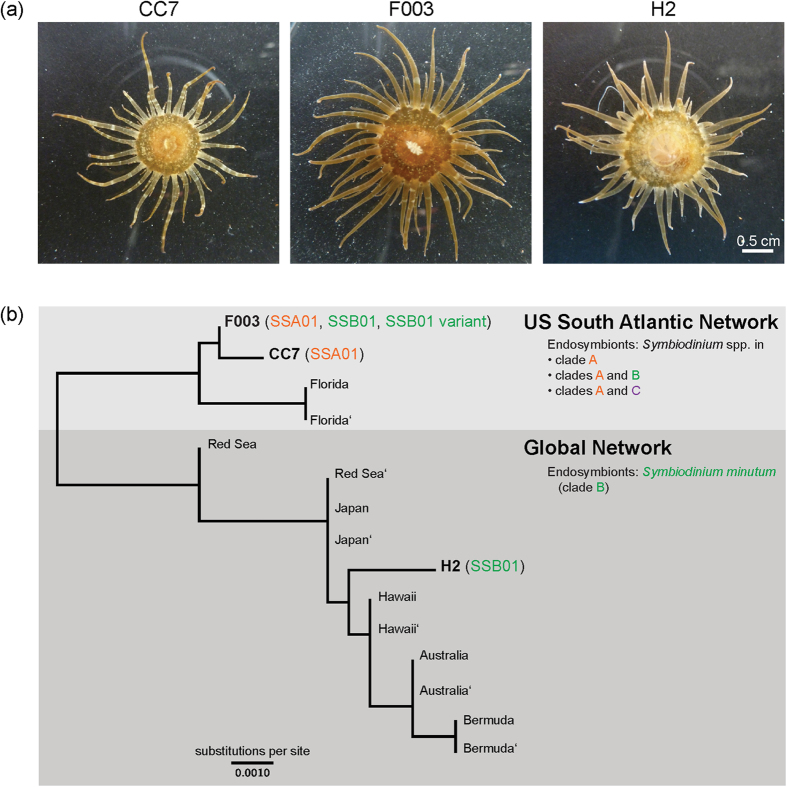
Characterization of *Aiptasi*a clonal lines CC7 (male), F003 (female), and H2 (female). (**a**) Clonal lines appear morphologically similar, with minor variation in coloration and body size. (**b**) Maximum likelihood tree of ~2 kb concatenated *SCAR* genotyping markers^24^ showing the relationship of the three clonal lines to twelve field-sampled *Aiptasia* sp. individuals^24^ grouped into two genetic networks. Clonal lines are indicated by name; field-sampled individuals are named by their sampling location, with the second animal from the same location designed by’. Shown also are the endogenous *Symbiodinium* spp. (clades A-C and strain names) hosted by *Aiptasia* in the given genetic networks^24^ and in the three laboratory clonal lines^22^,^26^; F003 symbiont characterization from this study.

**Figure 3:**
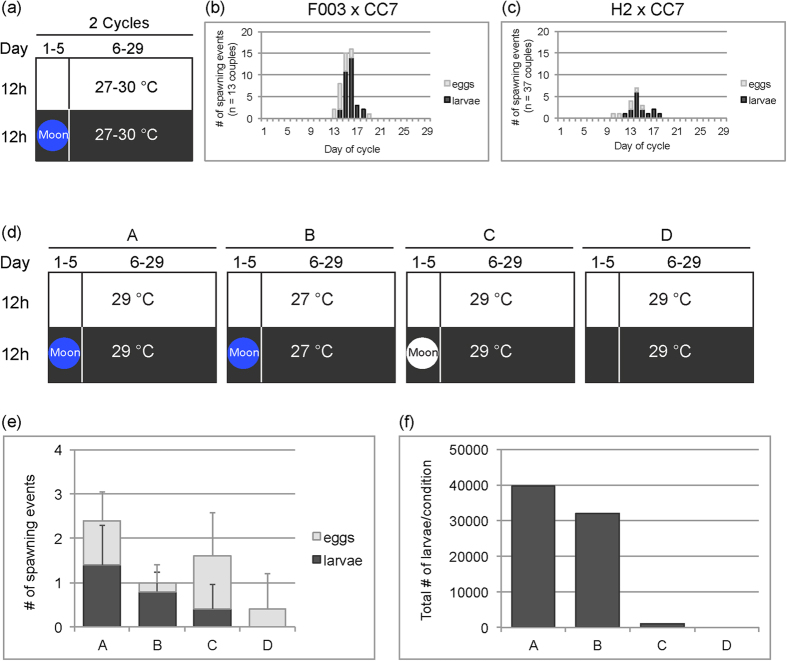
Induction of spawning in *Aiptasia.* (**a**) Schematic of initial spawning induction conditions during two consecutive 29-day artificial lunar cycles. Temperature and moon cue indicated; color of the circle refers to color of the simulated moon cue (see text). (**b**,**c**) Spawning synchronicity and efficiency in [F003 x CC7] couples (**b**) and [H2 x CC7] couples (**c**) under the induction conditions in (**a)**.(**d**) Schematics of various spawning induction conditions A-D for a direct comparison of spawning cues in [F003xCC7] couples. (**e**) Number of spawning events by five couples in each of the four induction conditions in (**d)** over one 29-day artificial lunar cycle. Error bars are standard deviations. (**f**) Quantification of total larvae per condition produced during the larvae spawning events shown in (**e**).

**Figure 4:**
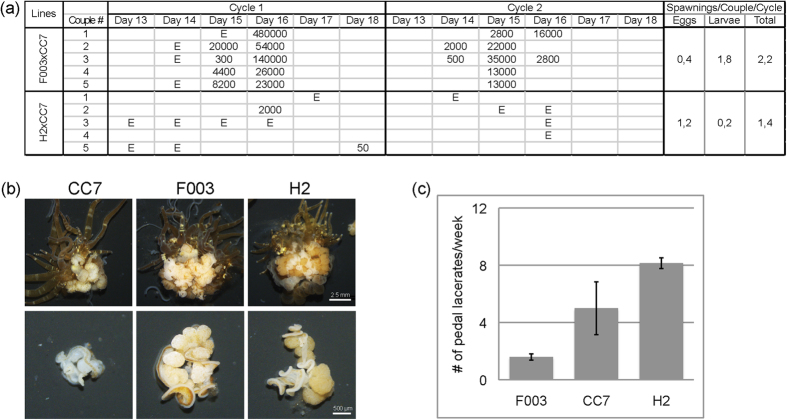
Line-specific differences in reproduction between *Aiptasia* female clonal lines. (**a**) Quantification of larvae and egg production by [F003 x CC7] and [H2 x CC7] couples in two cycles of spawning induction in condition A from Fig. 3d. E denotes occurrence of eggs; numbers indicate approximate quantification of larvae. (**b**) Cross-sections of longitudinally dissected anemones from the three clonal lines shows many bulbous gonads within mesentery tissues. (**c**) Weekly quantification of asexual reproduction via pedal laceration in the three clonal lines over five weeks. n = five individuals per clonal line; error bars are standard deviations.

A previous study of a wild clonal *Aiptasia* sp. population found no apparent tradeoff between reproductive modes, yet it was postulated that genotypes may allocate resources between reproduction modes differently, perhaps as an adaptation to the local environment^43^. To assess whether such tradeoffs or differences occur in various genotypes of *Aiptasia*, particularly in light of the observed differences in sexual reproduction (see above), we monitored the asexual reproduction rates of clonal lines CC7, F003, and H2. Asexual reproduction is achieved via pedal laceration^25^, wherein symbiont-containing lacerates bud off the pedal disc and migrate away from the parental animal over the course of several days, after which they develop tentacles (Fig. 1b). We quantified the number of pedal lacerates (pl) produced per animal per week and found that under standard growth conditions, H2 produces significantly more pedal lacerates (8 pl/week) than F003 (1.7 pl/week) (Fig. 4c). Male clonal line CC7 produces on average 5 pl/week (Fig. 4c). Thus, the rates of asexual reproduction differ greatly between genotypes with no clear correlation to the sex of the clonal line; within females, H2 asexually reproduces faster than F003 in these conditions.

## Discussion

Here we have developed a robust protocol to induce spawning in three clonal lines of the model symbiotic cnidarian *Aiptasia*, thereby opening new possibilities in analysis of symbiosis establishment, early development, and other key processes. Similar to the model cnidarian *Nematostella*, we find that extensive feeding of *Aiptasia* over multiple months in low-density culture is an important prerequisite for subsequent efficient spawning, especially for females. However, in resemblance to reef-building corals rather than to *Nematostella*, we have identified the key trigger to induce the synchronous release of gametes in well-fed *Aiptasia* animals to be the simulation of full moon by blue light during five nights within an artificial lunar monthly cycle. Interestingly, the wavelength of the applied light is consequential: LEDs exclusively emitting light between 400–460 nm effectively stimulate the release of gametes, while LEDs with white light, even when enriched in the blue wavelengths (such as actinic spectra) have minimal effect. This indicates that *Aiptasia*, similar to reef-building corals, may have specific blue-light-sensing photoreceptors (e.g. cryptochromes) that are thought to mediate synchronous mass spawning events of corals worldwide^40^,^41^. Indeed, we identified in the *Aiptasia* genome^28^ a homolog of the *Acropora millepora* blue-light cryptochrome receptor *cry2* (data not shown) that is expressed in correlation with coral spawning^41^. Many reef-building corals release gametes synchronously after the increase in water temperature (occurring during spring) in correlation to the last full moon and sunset^39^. Accordingly, we observed that a modest temperature shift (+3 °C) is not sufficient to induce efficient spawning in *Aiptasia* alone, but in combination with the simulation of full moon appears to be beneficial. However, *Aiptasia* collected in the wild has been proposed to spawn year-round in correlation with the lunar cycle^35^, suggesting no strict inherent temperature limitations, although influences of seasonal changes in spawning efficiency cannot be ruled out^36^. Taken together, our data support the idea that the blue light is the key spawning cue with minor influences of water temperature.

In studies of gonad development of *Aiptasia* populations in the wild, Chen and colleagues found that *Aiptasia* eggs appeared to be released about 8–14 days after the natural full moon^35^. In the studies herein, we observe the synchronous release of gametes on Day 13–17 within a 29-day cycle; because our simulated full-moon cues occurs on Days 1–5, this spawning corresponds to 8–12 days after the moon cue (i.e. peak of moonlight followed by increasingly darkening conditions). Thus, our studies match up remarkably well, with the timing of gamete release nearly identical between natural *Aiptasia* populations and our populations kept in the laboratory for over five years and stimulated by an artificial light cue. The persistence of this innate ability to respond to the stimuli indicates that synchronized spawning is strongly genetically encoded and shared worldwide throughout *Aiptasia* sp. including across the major global genetic networks. Moreover, we observe that *Aiptasia* gametes are released a few hours after diurnal onset of darkness (data not shown), which is likewise identical to reef-building corals that spawn a few hours after sunset, varying by species^45^,^46^. Taken together, the monthly timing (i.e. new moon) and diurnal timing (i.e. after sunset) ensure spawning during a period of the darkest possible conditions. This is hypothesized to not only aid in predation avoidance but also to ensure embryos pass through fragile cleavage stages prior to exposure to potentially damaging UV radiation during daylight^47^.

Interestingly, we find that the two female lines F003 and H2 differ in their reproductive strategies: under standard growths conditions and extensive feeding, the H2 colony expands more efficiently by asexual pedal laceration than F003, which in turn appears to invest a major part of incoming nutrients into oocyte production. The consistency of these responses indicates that they stem from genetically encoded differences^24^,^48^. It has been hypothesized that distinct genotypes with different allocation ratios between asexual and sexual reproduction may be favored in different habitats^49^. Consistent with the high asexual reproduction rates in H2, Thornhill and colleagues^24^ propose that efficient clonal propagation and associated vertical symbiont transmission of members of the global *Aiptasia* network primarily led to its worldwide distribution and status as an invasive species in aquaria and in the wild^35^,^50^,^51^, and the low-level genetic population structure of its associated endosymbiont *S. minutum*. Such extensive distribution therefore likely occurred quickly, via anthropogenic vectoring such as ballast waters, fouling of ships, aquaculture, or the aquarium trade^24^. In contrast, its lower asexual reproduction rates apparently restrict F003 to a more local distribution, yet its enhanced sexual reproduction rates would increase the genotypic diversity of its community. Indeed, this is recapitulated in the diversity of the sequenced *SCAR* markers: we found that F003 and CC7 showed greater genotypic diversity, with two alleles at several loci, whereas H2 was monoallelic at all four loci investigated. Such genotypic diversity might lead to the observed greater flexibility with associated symbionts^24^, and indeed we find that F003 hosts multiple endogenous *Symbiodinium* strains from different clades.

This study opens the door to a steady supply of *Aiptasia* larvae for a number of crucial purposes, not least of which is addressing what may be considered one of the final steps in the establishment of the *Aiptasia* model system: closing the life cycle in the laboratory via metamorphosis and settlement of planula larvae. To date this has not been reported, although metamorphosis and settlement under laboratory conditions has been established for corals^52^,^53^,^54^, providing vital guidance on how to approach this in *Aiptasia*. The protocol provided here allows sufficient *Aiptasia* larvae to systematically test and thereby identify metamorphosis and settlement cues in the laboratory. Although larvae production allows new techniques to be developed and important biological questions to be addressed in areas like development, endosymbiosis establishment and photobiology, microscopic imaging, gene knockdown, and transformation, settlement will further this by opening other paths like classical genetics and generation of transgenic lines.

These developments will bring *Aiptasia* fully into the stable of cnidarian laboratory models, which have proven to be vital in gaining basic biological insights. Yet *Aiptasia* also represents a new type of model system because of its additional feature of an ecologically relevant endosymbiosis. That photosynthetic symbioses appear in organisms across the tree of life makes comparative analyses of these critical relationships interesting in evolutionary and ecological contexts. The symbioses inherent in nearly all higher metazoans (including humans) have been increasingly recognized in the molecular age, and particularly in the global context of accelerating environmental change, *Aiptasia* will contribute to opening new paths in emerging fields that seek to describe the ever-more-recognized complexity of organisms in a changing world.

## Methods

### *Aiptasia* clonal lines

The founder animals for male line CC7^26^ and female line F003 (courtesy of the John Pringle lab) were originally collected by Carolina Biological Supply Company (#162865; Burlington, USA) from an area approximately centered on Wilmington, North Carolina, USA. The founder animal for line H2 was collected off Coconut Island, Hawaii^22^.

### Genotypic characterization of *Aiptasia* clonal lines

To genotypically characterize the three clonal lines CC7, F003, and H2, we used the *SCAR* markers developed by Thornhill and colleagues^24^. Genomic DNA was extracted from two representative animals of each line with the DNeasy Blood and Tissue Kit (#69504; Qiagen, Venlo, Netherlands) according to the manufacturer’s protocol for animal tissue. The gDNA of each animal was then amplified with each of the four *SCAR* marker primer pairs^24^: *ApSCAR3F* and *ApSCAR3R*; *ApSCAR4F* and *ApSCAR4R*; *ApSCAR5F* and *ApSCAR5R*; *ApSCAR7F* and *ApSCAR7R2*. Amplification reactions of 50 μL contained 2 U *Taq* polymerase, 0.2 μM of each primer, 200 μM dNTPs, 20 mM Tris-HCl (pH 8.8), 10 mM (NH_4_)SO_4_, 10 mM KCl, 2 mM MgSO_4_, 0.1% Triton^®^X-100, and 150 ng gDNA template. Amplification conditions were as follows: initial denaturation at 95 °C for two min; 30 cycles of denaturation at 95 °C, annealing at 58 °C for 45 s, extension at 60 °C for 1.5 min; final extension at 68 °C for 5 min. Products were purified with the Wizard SV Gel and PCR Clean-Up System (#A9281; Promega, Madison, USA), cloned, and four or more positive bacterial clones were sequenced with the M13F (−21) or M13R (−26) universal primers.

Sequences were processed in Geneious version 6.1.8^55^ [ http://www.geneious.com] by initially aligning to the reference sequences from Thornhill *et al.* and trimming and concatenating accordingly^24^. In cases where a given marker in an anemone line produced two alleles (see Results), the alleles were collapsed to create a consensus sequence containing the appropriate base ambiguity. The three clonal line sequences and the twelve reference sequences from Thornhill *et al.*^24^ were aligned with ClustalW^56^ and a maximum likelihood phylogenetic tree was generated using PhyML^57^ with default parameters and the general time-reversible (GTR) nucleotide substitution model. Tree topology searches chose the best topologies of both Nearest Neighbor Interchanges (NNI) and Subtree Pruning and Regrafting (SPR) topological moves. Topology, branch length, and substitution rate were optimized, and branch support was estimated by bootstrap analysis of 100 replicates.

### Identification of endogenous *Symbiodinium* in F003 clonal line

To determine which symbiont strain(s) are endogenous to the *Aiptasia* F003 line, we used the *Symbiodinium Domain V chloroplast large subunit ribosomal DNA* (*cp23S*) marker for phylogenetic analysis as previously described^22^,^58^. Three adult individuals were each homogenized with a MICCRA D1 homogenizer (Miccra, Müllheim, Germany) and algal genomic DNA was extracted using the DNeasy Plant Mini Kit (#69104; Qiagen, Venlo, Netherlands) following the manufacturer’s instructions. The *cp23S* region was amplified from each gDNA sample with the *23S4F* and *23S7R* primers^58^. Amplification reactions of 50 μl contained Phusion polymerase, 0.1 μM of each primer, 200 μM dNTPs, 1X Phusion HF buffer (#B0518S; NEB, Ipswich, USA), and ∼50–100 ng gDNA. Amplification conditions were as follows: initial denaturation at 94 °C for 5 min; 35 cycles of 94 °C for 30 s, 50 ˚C for 1 min, 72 °C for 1.5 min; and final extension at 72 °C for 5 min. For each amplification reaction, products (∼650 bp) were purified with the Wizard SV Gel and PCR Clean-Up System, cloned, and 18 or more positive bacterial clones were sequenced with the M13F (−21) universal primer. In total, 60 high quality *cp23s* sequences (18, 21, and 21 sequences per animal) were aligned in Geneious version 7.1.7^55^ [ http://www.geneious.com] and compared to *cp23S* sequences from SSB01^22^ (GenBank Accession #JX221048.1) and the CC7 endogenous clade A strain^26^ SSA01 (GenBank Accession #KT186239), which was sequenced as described above.

### *Aiptasia* culture conditions

General anemone stocks were maintained at a density of 30–40 animals (unless stated otherwise) per tank in medium-sized food-grade translucent polycarbonate tanks (GN 1/4–100 cm height, #44 CW; Cambro, Huntington Beach, USA) filled with artificial seawater (ASW) (Coral Pro Salt; Red Sea Aquatics Ltd, Houston, USA) at 31–34 ppt salinity. Stock tanks were kept in Intellus Ultra Controller Incubators (Model I-36LL4LX; Percival, Perry, USA) at 26 °C on a diurnal 12L:12D cycle (12 h light:12 h dark) under white fluorescent bulbs with an intensity of ~20–25 μmol m^−2^ s^−1^ of photosynthetically active radiation (PAR), as measured with an Apogee PAR quantum meter (MQ-200; Apogee, Logan, USA). Animals were fed two to five times per week with freshly hatched *Artemia* nauplii. Seawater in the tanks was exchanged two to three times per week and tanks were cleaned with cotton-tip swabs as required.

### Spawning induction of *Aiptasia*

To induce *Aiptasia* gamete release following a simulated full moon cue, individuals with oral disk diameter ~0.7 cm or greater were combined into couples ([H2xCC7] or [F003xCC7]), transferred to ASW in small food-grade translucent polycarbonate tanks (GN 1/9–65 cm height, #92 CW; Cambro, Huntington Beach, CA, USA) and kept in standard cell culture incubators (Heracell 150i; Thermo Scientific, Waltham, USA) at 27 *°*C–30 *°*C (see temperatures indicated in Results). Tanks were fed *Artemia* nauplii five times per week and kept on a diurnal 12L:12D cycle at intensities of 20–30 μmol m^−2^ s^−1^ provided by white LED lights, which were comprised of two types of LEDs—those with wavelengths of 425 nm–705 nm (color temperature 15,000 Kelvin) and those with wavelength 460 nm – at a ratio of 5:1 respectively (SolarStinger SunStrip “Marine”; Econlux, Cologne, Germany). A 29-day lunar cycle was simulated by subjecting the tanks to constant blue light during the 12 h “dark” period in the first five nights of the cycle (Day 1–5). Blue LED lights were comprised of LEDs with wavelengths 400 nm, 420 nm, 440 nm, and 460 nm at a ratio of 1:1:1:1 (SolarStinger SunStrip “Deep Blue”; Econlux, Cologne, Germany) and were 10–16 μmol m^−2^ s^−1^ in intensity. The presence of gametes or planula larvae was checked by examining the tanks with a Leica S8APO stereoscope.

### Isolation of larvae for quantification

Larvae were isolated from the spawning tanks by stepwise filtration: tank contents were first passed through a 70 or 100 μm cell strainer to withhold coarse particles and permit larval passage, after which larvae were concentrated by passage through a 40 μm cell strainer and then rinsed into glass beakers with filter-sterilized ASW. For approximate quantification, beakers were mixed well to evenly distribute larvae and six 10 μl drops were pipetted onto a glass slide. The average number of larvae per drop was used to extrapolate the approximate total.

### Dissection of gonads and gametes from *Aiptasia*

To promote gonad growth in anemones, 20–25 small anemones per clonal line (~0.5 cm height and no apparent gonads) were taken from stock tanks and combined into a medium-sized food-grade translucent polycarbonate tank, with two tanks per line. Animals were kept at 27 °C, fed five times per week with *Artemia* nauplii, and sampled after seven months. Anemones were transferred to a standard petri dish containing ASW and dissected in half longitudinally with a scalpel; mucus, acontia, and remaining food particles were removed with forceps. Animals were transferred to new petri dishes with ASW and images were captured using a Leica S8APO binocular (top illumination) equipped with a Leica MC170 HD color camera. Individual gonads were then detached from the anemones with forceps, transferred to new petri dishes, and imaged under identical parameters as above but with a higher magnification.

To isolate and image gametes, small fractions of male and female gonads in ASW were opened with scalpel and forceps. Gonads were placed by forceps (female) or pipette (male) onto glass microscope slides and covered with glass coverslip. Representative images were taken with a Nikon Eclipse 80i microscope equipped with a Nikon DS-1QM monochrome camera (sperm) or a Nikon DS-1U color camera (female gonad).

### Quantification of asexual reproduction rates

To determine the rate of asexual reproduction, five animals approximately three to four months old from each clonal line were individually placed into ASW in small food-grade translucent polycarbonate tanks and fed five times per week with *Artemia* nauplii. Pedal laceration was analyzed for each individual at the end of every week for five consecutive weeks. The number of pedal lacerates were counted by eye or with a stereoscope (Leica S8APO), and after counting all pedal lacerates were removed.

## Additional Information

**How to cite this article**: Grawunder, D. *et al.* Induction of Gametogenesis in the Cnidarian Endosymbiosis Model *Aiptasia* sp. *Sci. Rep.* **5**, 15677; doi: 10.1038/srep15677 (2015).

## Supplementary Material

Supplementary Information
