# Supplementary material for: Induction of Gametogenesis in the Cnidarian Endosymbiosis Model Aiptasia sp
Source: Sci Rep. 2015 Oct 26;5:15677. doi: 10.1038/srep15677 (PMC4620495; doi:10.1038/srep15677)
Supplement: Supplementary Information [file srep15677-s1.pdf]

SUPPLEMENTARY INFORMATION

INDUCTION OF GAMETOGENESIS IN THE CNIDARIAN ENDOSYMBIOSIS MODEL *AIPTASIA* SP.

Authors: D. Grawunder, E. A. Hambleton, M. Bucher, I. Wolfowicz, N. Bechtoldt, A. Guse

Lines	Couple #	# of months/condition		Tp	Cycle 1											Cycle 2										
		FOO3	CC7		Day 10	Day 11	Day 12	Day 13	Day 14	Day 15	Day 16	Day 17	Day 18	Day 19	Day 20	Day 10	Day 11	Day 12	Day 13	Day 14	Day 15	Day 16	Day 17	Day 18	Day 19	Day 20
F003 x CC7	1	4 *	3 *	27						L										E	L	L				
	2			27						E										E	E	E				
	3			30					E	L	L	L							E	L						
	4			30							L									E						
	5	3 *	3 *	29					E												L					
	6			29						L	L										L	L	L			
	7			29						E																
	8	5	3 *	29							L											L				
	9			29						L	L											L				
	10			29						L	L										L	L				
	11			29							L											L				
	12	8 *	2 *	27						E	L											L	L	L	E	
	13			29				E	L	L										E	L			L		
H2 x CC7	1	3-5 *	6 *	27					L	L										L						
	2			27					L								E									
	3			27					L																	
	4			27					L	L																
	5			27					L							E										
	6	3 *	3 *	29																						
	7			29																						
	8			29																						
	9			29																						
	10			29																						
	11			29																						
	12	4 *	4 *	29					E																	
	13			29																						
	14			29																						
	15	5 *	3 *	29																						
	16			29																						
	17			29																						
	18			29																						
	19	2 *	2 *	27						E																
	20			27																						
	21			27																						
	22			29				L																		
	23			29																						
	24			29				E																		
	25			29				L	L																	
	26			29																						
	27			29				E																		
	28	5 *	4 *	29																						
	29			29																						
	30			29																						
	31			29								L												L		
	32			29																						
	33			29																						
	34			29																						
	35			29							L	L														
	36			29																						
	37			29																						

Supplementary Figure S1. Data of initial spawning induction of *Aiptasia* clonal lines (Fig. 3a-c). Thirteen couples of [F003 x CC7] and thirty-seven couples of [H2 x CC7] were followed for two months under a simulated lunar cycle. E = eggs; L = larvae. Some couples were simultaneously exposed to other conditions to test whether these affected spawning success (see Results); # of months/condition is the duration in higher temperature and higher feeding conditions prior to spawning induction; Tp indicates temperature; * indicates exposure to faint blue light (~6 µmol m^{-2} s^{-1}) during night periods prior to the spawning induction.

Authors: D. Grawunder, E. A. Hambleton, M. Bucher, I. Wolfowicz, N. Bechtoldt, A. Guse

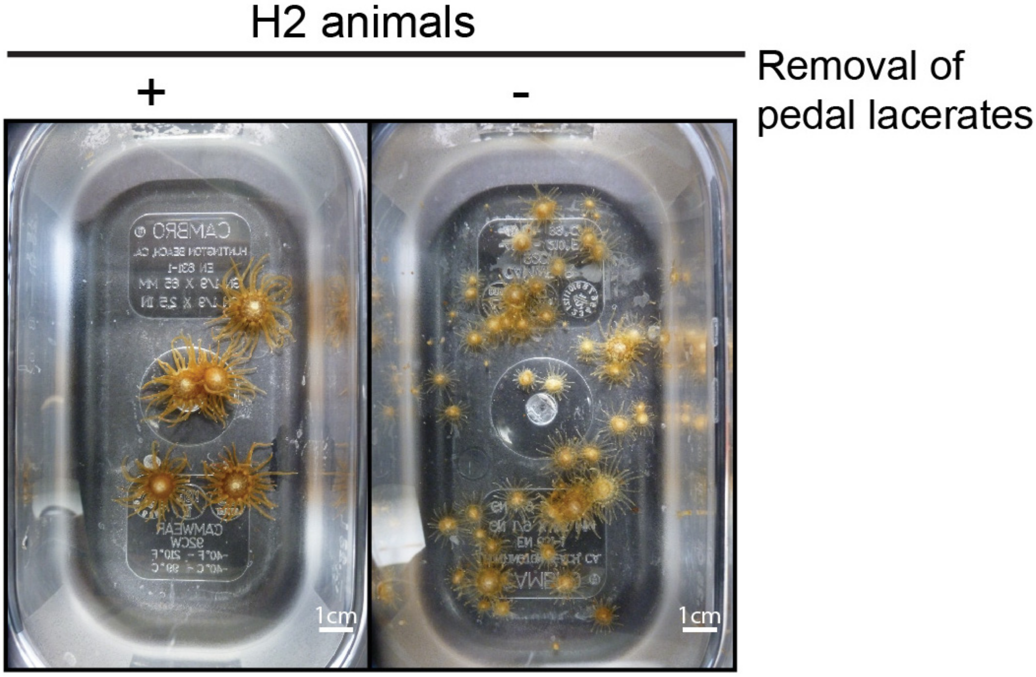

Supplementary Figure S2. Removal of pedal lacerates greatly promotes growth of individual adults. Five small adults from clonal line H2 were placed into ASW in small food-grade translucent polycarbonate tanks; two such tanks were created and kept under standard culture conditions. In the left-panel tank, all pedal lacerates were removed three times per week; in the right-panel tank, all pedal lacerates were left undisturbed. Tanks were imaged with a hand-held digital camera (Panasonic Lumix DMC-TZ18) after 7 months.

INDUCTION OF GAMETOGENESIS IN THE CNIDARIAN ENDOSYMBIOSIS MODEL *AIPTASIA* SP.

Authors: D. Grawunder, E. A. Hambleton, M. Bucher, I. Wolfowicz, N. Bechtoldt, A. Guse

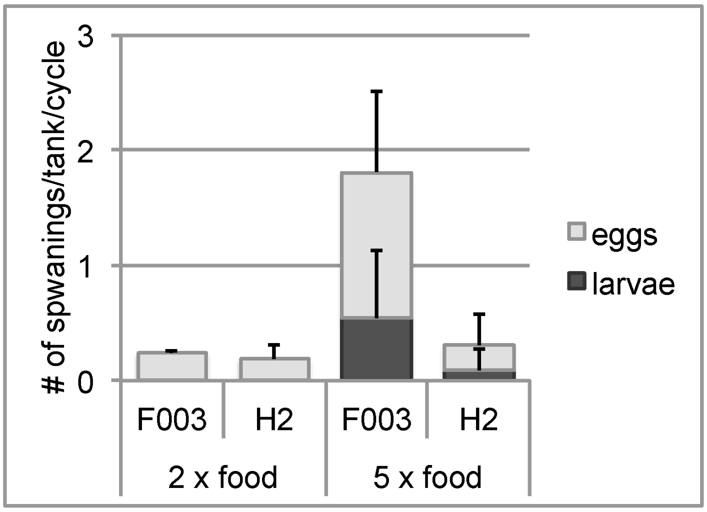

Supplementary Figure S3. Comparison of spawning efficiency before and after increased feeding shows a marked improvement in spawning, especially by [F003 x CC7] couples. Error bars are standard deviations.

INDUCTION OF GAMETOGENESIS IN THE CNIDARIAN ENDOSYMBIOSIS MODEL *AIPTASIA* SP.

Authors: D. Grawunder, E. A. Hambleton, M. Bucher, I. Wolfowicz, N. Bechtoldt, A. Guse

Condition	Couple #	Cycle 1									Spawnings/Couple/Cycle		
		Day 13	Day 14	Day 15	Day 16	Day 17	Day 18	Day 19	Day 20	Day 21	Eggs	Larvae	Total
A	1		L	15000									
	2	E	E										
	3	E	L	5000							1,0	1,4	2,4
	4	E (few)	L	2800									
	5		E	17000									
B	1			8000									
	2			200									
	3	E		7300							0,2	0,8	1,0
	4												
	5			16500									
C	1												
	2				E (few)	50			E	E			
	3								E (few)		1,2	0,4	1,6
	4				E (few)	1250							
	5								E (few)				
D	1												
	2												
	3										0,4	0	0,4
	4		E (few)	E (few)									
	5												

Supplementary Figure S4. Data of direct comparison of spawning induction conditions using five couples of [F003xCC7] per condition (Fig. 3d-f). Couples were followed for one 29-day artificial lunar cycle. E = eggs; L = larvae.

INDUCTION OF GAMETOGENESIS IN THE CNIDARIAN ENDOSYMBIOSIS MODEL *AIPTASIA* SP.

Authors: D. Grawunder, E. A. Hambleton, M. Bucher, I. Wolfowicz, N. Bechtoldt, A. Guse

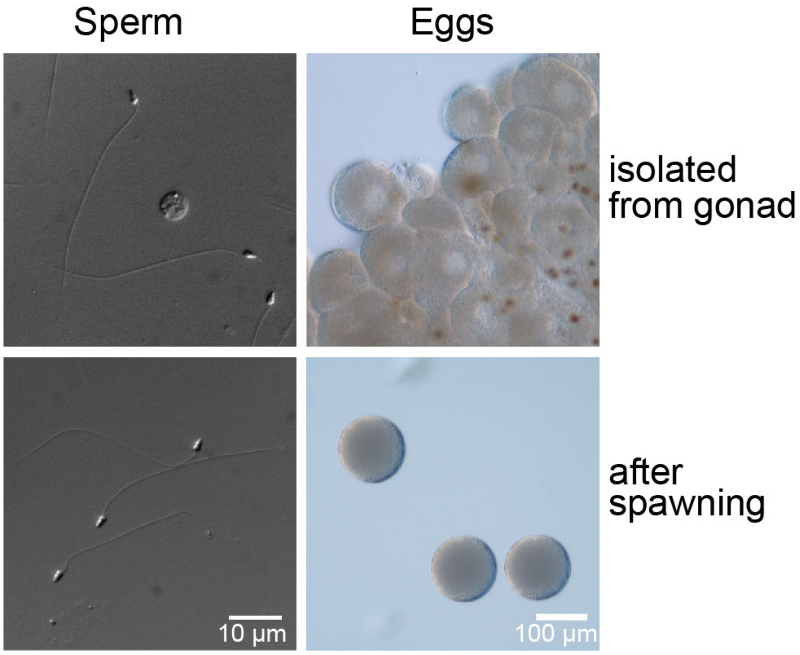

Supplementary Figure S5. Male and female gametes isolated from gonads or after spawning.
